# AI Chatbot Answers for Drug Dosing Adjustments According to Renal Function in Geriatric Patients Using the New Scoring System (AI Quality Output Score): Cross-Sectional Study

**DOI:** 10.2196/87803

**Published:** 2026-06-05

**Authors:** Celine Barbonus, Ralf Sultzer, Thilo Bertsche

**Affiliations:** 1 Department of Clinical Pharmacy, Institute of Pharmacy, Faculty of Medicine, Leipzig University, Brüderstraße 32, Leipzig, Saxony, 04103, Germany, 49 3419711800; 2 Drug Safety Center, Leipzig University and University of Leipzig Medical Center, Leipzig, Saxony, Germany; 3 Sana Geriatric Centre Zwenkau, Zwenkau, Saxony, Germany

**Keywords:** artificial intelligence, AI, large language models, LLMs, pharmaceutical, score, decision-making

## Abstract

**Background:**

Preventable adverse drug reactions in geriatric patients are caused by overdosing, especially in cases of impaired renal function. Artificial intelligence (AI) chatbots are being discussed as tools to generate drug information, which can adjust drug dosing and prevent subsequent adverse drug reactions based on individualized patient data. However, the question arises as to the extent to which such AI chatbots can withstand scientific evaluation in this task.

**Objective:**

We newly developed and validated the AI quality output score (AQUOS, ranging from 0% to 100%) to assess the quality of AI chatbot answers. We investigated whether AQUOS depends on (1) renal function, (2) medication complexity, (3) prompting language (English and German), and (4) whether the answers are reproducible (assessed at 2 independent times). Additionally, we assessed the potential for harm.

**Methods:**

In a standardized prompt, we asked 4 AI chatbots (ChatGPT, Copilot, Gemini, and Scite) whether the medication of 100 geriatric patients with polymedication at discharge should be adjusted according to their renal function. We prompted drug-related queries in 2 languages and at 2 times to assess AI chatbot answers, and we scored the generated outputs based on AQUOS. Additionally, we assessed possible harm from the AI chatbot answers using the World Health Organization definition “The conceptual framework for the international classification for patient safety.”

**Results:**

We analyzed 1600 AI chatbot answers, with AQUOS values ranging from −19.0% to 95.2%, depending on the chatbot. We found that AQUOS declined with decreasing renal function (ChatGPT: −0.215; *P*=.03) and increasing medication complexity (Scite: −0.239; *P*=.02). Possible harm also correlated with more complicated patient statuses (lower kidney function and higher medication complexity) across all chatbots. Overall scores were up to 4.8% higher in English than in German prompting. The AI chatbot answers were highly reproducible.

**Conclusions:**

In renal drug dosing, the quality of AI chatbot answers declined as renal function decreased and medication complexity increased. Even the highest AQUOS achieved is insufficient for deploying AI chatbots in the high-risk health care sector.

## Introduction

Artificial intelligence (AI), and especially AI chatbots, has become important in medical practice, supporting clinical decision-making [1,2]. AI chatbots, AI-based search engines, and other large language models (LLMs) are increasingly studied for their potential to make therapy processes—and, in particular, the gathering of drug information—faster and more efficient. In contrast to the high number of publications dealing with AI in health care, only a few involve real patient data [3]. Earlier studies often focus on limited aspects and lack practical relevance to specific clinical needs, such as renal dosage adjustment [4-6].

Adjusting medication based on renal function is challenging, especially for geriatric patients. Adverse drug reactions often result from overdosing, frequently caused by impaired renal function [7]. Age-related changes in pharmacokinetic parameters, comorbidities, and polypharmacy require careful, individualized drug therapy, especially in terms of patient safety and the safety of drug therapy [8,9]. However, tailored medication dosages adjusted to renal function should also be readily available under routine conditions to prevent avoidable adverse drug reactions. AI chatbots could have great potential in this context, although it should be noted from a legal perspective that they are not classified as medical devices.

Until now, no standardized score has existed for a quantitative quality assessment of AI chatbot answers in drug-related queries. To close this gap, we developed a new score to evaluate the quality (AI quality output score [AQUOS]) of AI chatbot answers based on the literature, adapted for drug-related queries [10,11]. Rather than relying on casual, everyday phrasing, prompts were structured using prompt engineering to provide consistent input and optimize AI potential [12,13]. Because medication queries are not only applicable to geriatric patients and renal function, AQUOS applies to a broader range of drug-related clinical scenarios.

Our study aimed to evaluate the quality and potential harm of drug-related queries addressing tailored renal dosing. We developed a score assessing quality (AQUOS) to see how it varies with renal function and medication complexity (ie, the number of drugs prescribed). Furthermore, we evaluated 2 prompting languages, as in routine practice, many questions are asked in the native language, and no previous studies have compared multilingual performance in this context. We also tested whether the AI chatbot outputs are reproducible over time. In addition to the score, we assessed potential harm according to the World Health Organization (WHO) definition.

## Methods

The cross-sectional, observational study is based on the CHART (Chatbot Assessment Reporting Tool) checklist (Checklist 1) [14].

### Ethical Considerations

The ethics committee of the Medical Faculty of Leipzig University (231/24-ek) approved the procedure on July 29, 2024. Due to the retrospective collection of patient data, no informed consent for publication was obtained; therefore, written informed consent was not required. Anonymized prompts were used to ensure patient privacy.

### Setting

In 2024, we used GeriDoc from the Geriatrics in Bavaria database to collect retrospective patient data from a geriatric hospital.

### Patient Data

We included data from geriatric patients in the rehabilitation ward who were hospitalized in 2023.

One hundred patients who meet the inclusion criteria must be included. An additional 10 patients were used for the pretesting of the AQUOS, and these patients were excluded from the main analysis.

### Inclusion Criteria

Eleven randomly selected patients per month from January 2023 to October 2023 were chosen for this study. To be included, renal function (glomerular filtration rate [GFR]) and polymedication (at least 5 drugs) at discharge had to be documented. In this case, polymedication means the patient must take at least 5 drugs; for example, a combined preparation of 2 drugs counts as 2, not 1.

### Study Design

In this study, we compared 4 different AI chatbots and designed structured inputs or prompts. After receiving ethical approval, we generated the AI outputs from October 18 to October 30, 2024, in Leipzig, Germany. The GFR was categorized into the typical 5 stages of renal disease: category 1 as normal renal function, category 2 as slightly reduced, category 3 as moderately reduced, category 4 as severely reduced, and category 5 as renal failure. The inputs were designed in both German and English, and outputs were evaluated at 2 time points: t0 and t1 (8 d later) to test reproducibility (not the learning effect after updates). Thus, t1 serves as the control for t0. We used an AQUOS (Multimedia Appendix 1) for evaluation. Additionally, discharge medications were categorized by complexity: low (5‐9 drugs), medium (10-14 drugs), and high (≥15 drugs). In this study, medication complexity is defined exclusively as complexity based on the number of medications and is divided into 3 categories (low, medium, and high). This measure reflects structural complexity based on the number of prescribed drugs and does not account for pharmacological risk, therapeutic drug classification, or potential drug-drug interactions.

### Prompting

We created a prompt based on prompt engineering, assigning roles to both the AI chatbot and the requester. We specified the renal function as GFR and listed the patient’s discharge medications and dosages. Following this, we instructed the chatbot to provide a precise answer if a dosage adjustment was necessary and to cite sources as a concrete task using both German and English prompts. The following prompting structure was developed based on literature [12,13] by the 2 authors, CB and TB, who are both pharmacists. We adjusted the prompt based on the quality of the AI chatbot’s responses. The aim was to frame the query as a standardized zero-shot prompt without any follow-up prompts. Neither patients nor members of the public were involved in the development process.

An example prompt in English:

*I am a physician in a hospital. Give your answers from a pharmacist’s point of view. It’s about a geriatric patient with a GFR of 62 ml/min who is taking the following medication: Acetylsalicylic acid 100 mg once a day, Ramipril 10 mg once a day, Atorvastatin 10 mg once a day, Pantoprazole 20 mg once a day, Metamizole 500 mg if required up to four times a day. Give a precise, short answer whether and how the dosage should be adjusted for the current GFR. Give reliable sources, including links, to your answer*.

The prompt was deliberately restricted to GFR, drug name, dose, and frequency. This standardized, minimal input format was chosen to ensure comparability across all 100 patient cases and all 4 AI chatbots and to reflect a realistic scenario of brief, point-of-care queries as they might occur in routine clinical practice. We acknowledge that clinically valid renal dosage adjustment may additionally depend on variables such as the indication for each drug, route of administration, treatment duration, dialysis status, body weight, or the differentiation of acute and chronic renal impairment. While more complex prompt formats incorporating additional clinical variables could yield more nuanced AI outputs, such designs would compromise standardization and cross-case comparability, which was an essential aspect of this study. The omission of these variables limits the clinical interpretability of the findings and should be considered when applying results to real-world settings.

If multiple GFR values were included in the discharge letter, the median GFR was used. The drug dosing was provided as in the example, along with the drug name and dosage frequency. We extracted chatbot outputs from the platform and saved them locally, starting a new conversation for each prompt while ensuring previous conversations were cleared. We configured the software to prevent it from “remembering” previous conversations. If a network error occurred, the output was regenerated. After generating the outputs, we verified that the companies had made no relevant updates to the AI chatbots.

Considering that the prompts were entered in German and English at 2 different times (t0 and t1) in 4 different AI chatbots each, 16 outputs were generated per patient.

### AI Chatbots

When collecting the AI chatbots, we focused on large, well-known AI chatbots. Moreover, appropriate settings had to be available to prevent entered data from being used for future AI model updates.

In terms of terminology, “AI chatbot” refers to the conversational interface accessed by users, “LLM” refers to the underlying language model architecture, and “model” is used as a general term for naming the different versions.

We used OpenAI GPT-4 (gpt-4o-2024-11-20, knowledge cutoff at October 01, 2023), Microsoft Copilot Business, Google Gemini 1.5 Flash (gemini-1.5-flash-002), and Research Solutions Scite, all of which are closed sources [15,16]. The exact models of the AI chatbots were not consistently accessible at the time of data collection, but we verified post hoc that no relevant model updates were released by the respective providers during the data collection window. We have accessed the AI chatbots using the web interface of each provider. Furthermore, the standard settings (eg, temperature) were applied to all AI chatbots, which were used as base models as provided by their respective companies. For each patient, a new conversation was initiated by entering the prompt and then saving the AI chatbot’s response locally, and the conversation was subsequently deleted before the next case was processed. With this procedure, no carry-over effects between patient cases could occur.

### Quality Score (AQUOS)

Based on previous research [10,17,18], we implemented an AQUOS (Multimedia Appendix 1) to evaluate the outputs of the AI chatbots. The score consists of 9 items. The first 5 items, rated from 0 to 4, focus on completeness, referencing, reference suitability, correctness of dosage recommendations, and dosing accuracy. The sixth item, rated 0 or 1 point, checks for disclaimers and references to health care professionals, patient monitoring, and the individual patient case. The last 3 items allow point deductions for unnecessary additional information, incorrect use of medical terms, and inappropriate language or phrasing, with greater issues leading to greater point reductions. Thus, the highest achievable score was 21, which equals 100%.

As a reference standard for evaluating the correctness of the AI chatbot outputs, the German database “Dosing” [19], combined with the corresponding summary of product characteristics, was used. In cases of discrepancies, the more recent source was given preference. If discrepancies persisted, clinical guidelines, original publications, or information from the European Medicines Agency were consulted. In Multimedia Appendix 2, some higher quality and poorer examples of AI chatbot outputs are provided.

### Assessment of Harm

In addition to AQUOS, the possible harm the chatbot could have caused with its response was assessed. The outputs were evaluated to determine whether the specific output could cause harm if provided to a physician as pharmaceutical advice. The possible harm was ranked using the *Conceptual Framework for the International Classification for Patient Safety* by the WHO Patient Safety [20]. The ranking of the harm ranges from “none” to “death,” according to the WHO categories: none, mild, moderate, severe, and death (0-5). Since the scaling differs from AQUOS, possible harm was considered separately.

### Validation Procedure of AQUOS

The validation of the AQUOS scoring system was conducted in 2 sequential phases, following the principles of internal and external validation as commonly applied in laboratory and clinical assay development.

This validation design used in this study broadly follows the principles used in the development of clinical scoring systems, where initial reliability testing is followed by a comparison with expert consensus, including an interrater reliability and intraclass correlation coefficient [21-25]. The detailed validation procedure is provided in Multimedia Appendix 3.

### Outcomes

We analyzed the points achieved in AQUOS to evaluate the quality of the AI chatbot outputs. Medication count–based complexity (categorized into 3 groups depending on the number of drugs, not the type of drugs or, eg, possible drug-drug interactions) and GFR (categorized according to the typical 5 stages, ranging from normal renal function to renal failure [26]) were analyzed in correlation with the score. Additionally, we investigated whether the AQUOS differs between English and German and whether the AI chatbot answers are reproducible over time (t0 and t1).

### Statistical Methods

To analyze the data, we conducted descriptive analyses (mean, median, relative difference, and correlations) and a paired, 2-tailed *t* test to assess statistical significance, with *P*<.05 considered statistically significant. To evaluate the first validation phase, we performed Cohen κ to examine whether the score was objective and to continue with just 1 rater in the study. For the second validation phase of AQUOS, we conducted an intraclass correlation coefficient analysis among the expert opinions, and AQUOS was then correlated with the median of the expert ratings using the Spearman’s correlation.

The statistical analysis was performed using IBM SPSS Statistics version 29 and Excel version 2408 from Microsoft 365.

## Results

### Overview

The main analysis included 100 geriatric patients, while the pilot study involved 10 geriatric patients. The mean (SD) number of discharge medications was 11.4 (4.2) for the primary group and 11.1 (2.8) for the pilot group. Patients’ characteristics and the medication count from the main analysis are summarized in Table 1.

**Figure 1.**
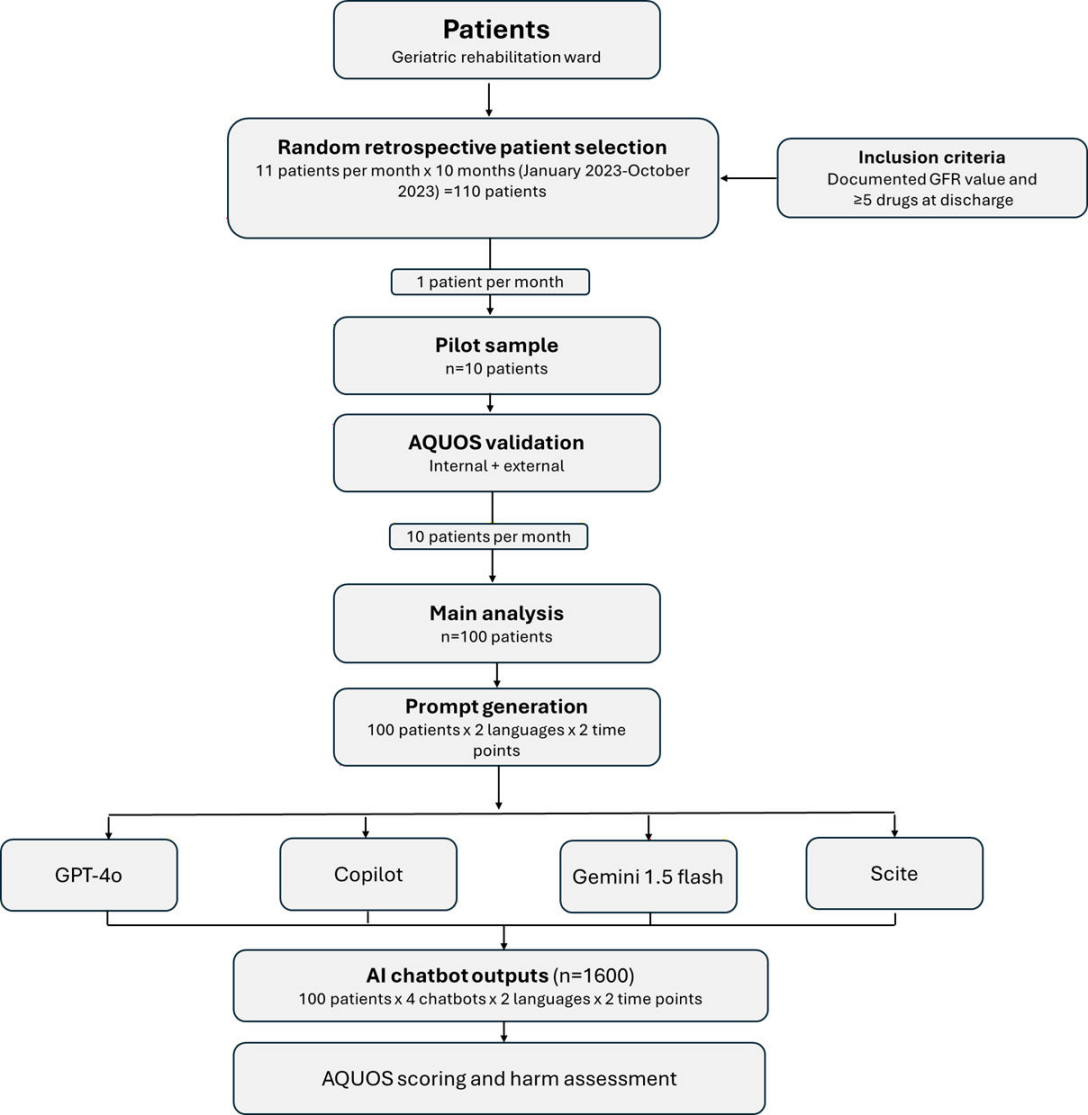
Study flow diagram. From 110 randomly selected geriatric patients (11 patients/mo, January 2023-October 2023), 10 were allocated to the pilot sample for artificial intelligence quality output score (AQUOS) validation, and 100 to the main analysis. The 100 patients included in the main analysis were each prompted across 4 AI chatbots, 2 languages, and 2 time points, resulting in 1600 outputs evaluated using AQUOS and World Health Organization (WHO) harm classification. GFR: glomerular filtration rate.

In total, we generated 1600 outputs using the AI chatbots. More precisely, this means that 16 outputs were generated per patient using 4 chatbots (ChatGPT [OpenAI], Copilot [Microsoft], Gemini [Google], and Scite [Research Solutions]), each at 2 different times for the investigation of reproducibility (t0 and t1) and in 2 different languages (German and English). Multimedia Appendix 2 provides examples of how the AI chatbots’ outputs were evaluated. A study flow diagram illustrating patient selection, the pilot sample, the derivation of the final 100 included patient cases, and the generation of 1600 AI chatbot outputs is provided in Figure 1.

**Table 1.** Patient characteristics (N=100; all geriatric patients) and medication count from the main analysis^a^.

Characteristics	Low complexity	Medium complexity	High complexity
Number of patients	33	46	21
GFR^b^, mean (SD; min-max)	67.9 (18.3; 28.0-109.0)	64.6 (18.1; 19.5-100.5)	61.2 (25.1; 15.0-97.0)

a Number of drugs taken in different complexity intervals: low complexity=5‐9 drugs, medium complexity=10‐14 drugs, and high complexity=at least 15 drugs. The renal function was measured as GFR.

b GFR: glomerular filtration rate.

### Validation of AQUOS

As a result of the first validation phase, the score was considered objective with a Cohen κ of 0.971, so there was 1 rater, a pharmacist, responsible for scoring. No patients or members of the public were included in the scoring process. For the second score validation phase, the intraclass correlation coefficient of 0.906 (95% CI 0.795‐0.974; *P*<.001) shows excellent agreement between the raters of the expert panel. In addition, the Spearman correlation, with a Spearman ρ of 0.650 (95% CI 0.012‐0.912; *P*=.04) between AQUOS and the median of the expert panel, validates AQUOS in an external validation conducted by experts.

### Renal Function

There were 5 patients in the normal renal function category, 62 in category 2 (slightly reduced), 25 in category 3 (moderately reduced), 8 in category 4 (severely reduced), and no patients in category 5 (renal failure) based on GFR classification.

**Figure 2.**
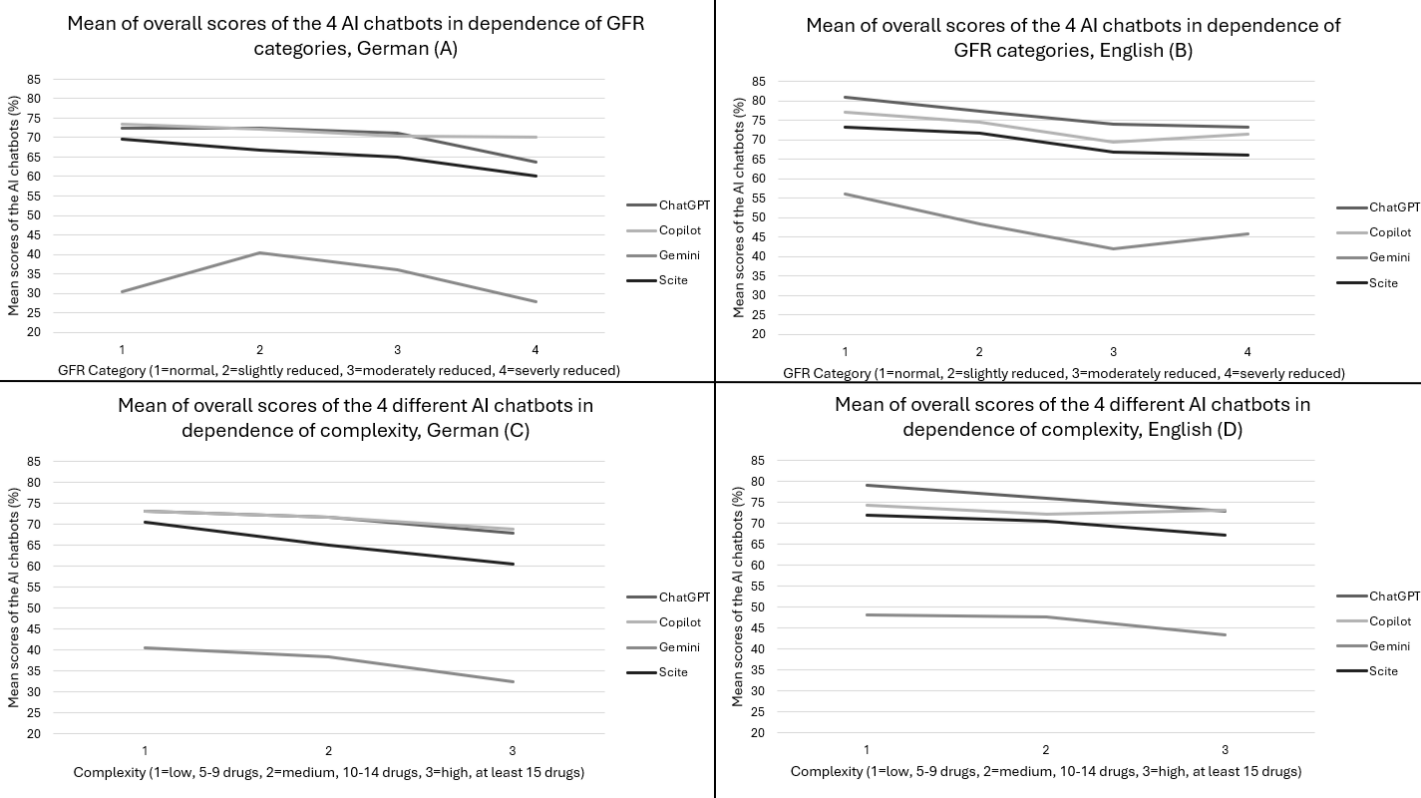
Mean overall scores (artificial intelligence quality output score [AQUOS]) of 4 AI chatbots based on glomerular filtration rate (GFR) categories (A, B) and the complexity of medication (C, D) in both German and English. AI: artificial intelligence.

The trends of overall AQUOS in German and English, as shown in Figure 2 and Multimedia Appendix 4, decline with worsening renal function (ChatGPT: –0.215, *P*=.03; Copilot: –0.258, *P*=.01; Scite: –0.357, *P*<.01; Multimedia Appendix 5). Regarding the English outputs, Copilot and Gemini had higher overall mean (SD) scores from category 3 (69.3%, 16.0% and 41.9%, 60.0%) to category 4 (71.4%, 9.4% and 45.8%, 31.4%). The maximum overall mean AQUOS was reached by ChatGPT (81.0%, SD 18.6%). Gemini had the lowest overall mean AQUOS of 41.9% (SD 60.0%). ChatGPT reached the highest single overall AQUOS with 95.2%, while Gemini achieved the lowest overall score in English (−19.0%).

Gemini caused possible mild harm in category 2 (mean 0.6, SD 0.9). In category 3, mild harm was noted in Copilot (mean 0.5, SD 0.7), Gemini (mean 0.7, SD 0.7), and Scite (mean 0.5, SD 0.7). Category 4 indicated overall possible mild harm across all chatbots. Significant correlations between possible harm and GFR categories were found: ChatGPT (0.396; *P*<.001), Copilot (0.476; *P*<.001), and Scite (0.443; *P*<.001).

### Medication Count–Based Complexity

In the low-complexity category, 33 patients were included; in the medium-complexity category, 46 patients were included; and in the high-complexity category, 21 patients were included.

The trends of overall AQUOS in German and English are shown in Figure 2, with specific values provided in Multimedia Appendix 6. The overall AQUOS declines as the complexity increases (Scite: −0.239, *P*=.02; Multimedia Appendix 5). Regarding the English outputs, the only exception is Copilot, where AQUOS increases from medium to high complexity, but not more than the score in low complexity. The maximum overall mean AQUOS was achieved by ChatGPT (79.1%, SD 9.0%). The lowest overall mean AQUOS was observed Gemini (43.3%, SD 25.5%). In low complexity, ChatGPT reached the highest single overall AQUOS (95.2%), while Gemini reached the lowest single overall score (−19.0%).

Gemini caused possible harm in medium complexity, with a mean of 0.9 (SD 1.0), corresponding to mild harm. In high complexity, possible harm was mild and was caused by Gemini (mean 0.6, SD 1.0). There was a significant correlation coefficient between the possible harm and the complexity only in German: ChatGPT (0.219; *P*=.03), Copilot (0.226; *P*=.02), and Scite (0.237*; P*=.02).

### Prompting Language

**Figure 3.**
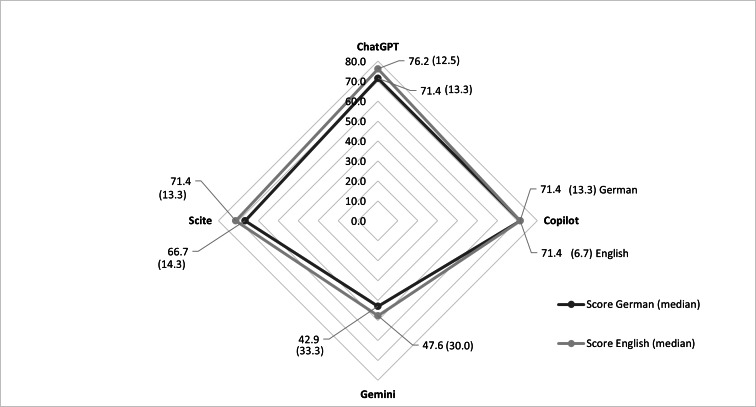
Comparison of the prompting languages, German and English, and 4 different AI chatbots (ChatGPT, Copilot, Gemini, and Scite), showing median overall scores (IQR) [%].

For every chatbot, the overall AQUOS were higher when the prompt was in English than in German (Figure 3). The only exception was Copilot, whose overall AQUOS scores were the same (median 71.4% [IQR 13.3%] in German and 71.4% [IQR 6.7%] in English) in both languages. ChatGPT achieved the highest scores in English (median 76.2% [IQR 12.5%]), while Gemini reached the lowest scores in German (median 42.9% [IQR 33.3%]). There was no possible harm in the median; however, regarding the IQR of Gemini in both languages, there was mild harm.

### Reproducibility

There was no significant difference between t0 and t1 regarding the overall AQUOS (Table 2). However, some single-score criteria showed significant differences.

In low complexity, there was a significant difference at Gemini in English regarding AQUOS criterion 6 (referral to medical or pharmaceutical professionals or follow-up checks), with a relative difference of 15.2% (*P*=.02). In medium complexity, there was a significant difference at ChatGPT in German for criterion 3 (suitability of the references given), with a relative difference of 5.4% (*P*=.01). Other significant differences were observed at Gemini, all in English, for criterion 4 (correct dose recommendation) and possible harm, with corresponding relative differences of 16.9% (*P*=.004) and 12.5% (*P*=.001).

**Table 2.** Reproducibility for all overall artificial intelligence quality output scores (AQUOS; t0 vs t1) and relative differences (*P* value)^a^.

Complexity	ChatGPT		Copilot		Gemini		Scite	
	German	English	German	English	German	English	German	English
Low complexity (%)	2.50 (*P*=.94)	1.89 (*P*=.17)	2.95 (*P*=.08)	2.42 (*P*=.51)	2.58 (*P*=.12)	5.33 (*P*=.72)	2.42 (*P*=.16)	1.52 (*P*=.34)
Medium complexity (%)	2.66 (*P*=.08)	2.23 (*P*=.66)	1.47 (*P*=.41)	2.07 (*P*=.94)	1.20 (*P*=.52)	6.03 (*P*=.42)	1.74 (*P*=.25)	0.82 (*P*=.66)
High complexity (%)	4.40 (*P*=.41)	2.98 (*P*=.23)	4.29 (*P*=.86)	1.43 (*P*=.43)	2.74 (*P*=.69)	4.29 (*P*=.68)	2.62 (*P*=.75)	2.14 (*P*=.37)

a Number of drugs taken in different complexity intervals: low complexity=5‐9 drugs; medium complexity=10‐14 drugs; high complexity=at least 15 drugs, and the 4 different chatbots (ChatGPT, Copilot, Gemini, and Scite).

## Discussion

### Key Findings

#### Overview

To evaluate AI chatbot outputs in drug-related queries, we developed the AQUOS (Multimedia Appendix 1). Using this score, we wanted to find out (1) how it changes depending on renal function, (2) the complexity of the medication taken by the patients, (3) the influence of the language used for prompting, and (4) whether the outputs are reproducible over time.

AQUOS declined with decreasing renal function and increasing medication count–based complexity, with possible harm correlating accordingly. Overall scores were higher in English than in German prompting, and AI chatbot answers were highly reproducible across both time points.

#### Renal Function

The poorer overall AQUOS with worsening renal function, alongside the correlation between lower GFR and increased potential harm, is consistent with findings by van Nuland et al [27], who similarly reported poor ChatGPT performance in patients with renal dysfunction. Contrary to this study, we found that GPT-4 reaches mean scores, and the single-score criteria correctness and accuracy (criteria 4 and 5) are always higher than 70% in English. So, even regarding the difficult comparison because of different methods, the quality of ChatGPT was better in our study. However, we also used a newer AI chatbot that could be better and more precise regarding renal function and dosage adjustments. But the pattern of AI chatbots performing poorly in queries from patients with renal dysfunction reflects the greater clinical complexity associated with this condition, which current AI chatbots appear less trained to handle. This may be due to under-representation of such patient populations in training data.

#### Medication Count–Based Complexity

AQUOS generally decreases with increasing medication count–based complexity, and patient safety was most favorable in the low-complexity group. Splitting complex medication lists into separate queries could be a practical interim approach, though this would preclude the assessment of drug-drug interactions and requires evaluation in future studies. This measure could solve this issue, together with more precise and advanced training of AI chatbots in drug-related queries, but it needs to be validated in further studies. However, in this design, the chatbot’s answers are more clinically useful and safer for the patient if the complexity is lower.

Roosan et al [6] investigated whether GPT-4 could solve patient cases of different complexity—here defined as difficulty—accurately in terms of drug interactions, the precision of recommendations and alternatives, and the adequacy of the created medication plans. They did not prompt in just 1 input but used a new prompt for each of the 3 key aspects. All 39 patient cases were solved correctly, with ChatGPT required to reach a threshold of 70% to be rated as correct.

#### Prompting Language

Because of the higher overall scores in almost all AI chatbots in English, likely due to the majority of training data being in English, the possible development of, for example, a medicinal product from an AI chatbot in the future suggests that the query should be in English, or it would be an option to translate the query. However, at this point, further research is necessary to determine the quality of the AI chatbot outputs when we instruct the chatbot to translate the prompt into English and then answer the query.

Jin et al [28] tested different languages (English, Hindi, Chinese, and Spanish) in LLMs and found that the best answers were given in English. However, they did not investigate German in this study. Schlicht et al [29] also examined German (in addition to English, Turkish, and Chinese) and tested GPT-4, among others, as we did. They found that there is quite a bit of variability between the languages and the associated outputs—for example, in output length or the consistency of information in the output of different languages.

Beyond quality differences, the observed language advantages in English raise important equity concerns. Many clinicians worldwide practice in non–English-speaking environments and will naturally query AI chatbots in their native language. If responses in languages other than English are generally of lower quality, this may compromise safe and effective use in these settings. As an interim solution, non-English queries could be translated during the query process into English, or validated translation tools could be used before prompts are entered, though the quality implications of such translated inputs require further evaluation. More generally, the English-centric focus of current training data for LLMs carries the risk of further worsening global inequalities in health care; therefore, institutions or regions with limited English knowledge may benefit less from AI tools in clinical environments and may be exposed to a higher risk. In the future development of AI tools, like AI chatbots, especially for clinical use, multilingualism should be explicitly considered as a key quality and safety criterion.

#### Reproducibility

Our good overall reproducibility across both time points differs from the results of Morath et al [5], who took 3 inputs at 4 different times. In their study, 3 out of 12 were the same over time, and no objective statistics were made. Furthermore, Al-Dujaili et al [18] tested 20 fictive patient cases using GPT-3.5, over 3 time points with a Cohen κ, finding a moderate positive agreement overall, which is in line with our findings. However, a comparison of their data to our findings is not valid, because we had 100 double-inserted inputs, used 4 different chatbots (including a different and more recent ChatGPT version), and analyzed the outputs using a scoring system, comparing the scores statistically with relative differences.

### AI Quality Output Score

In this study, we tested our AQUOS for the first time, and to our knowledge, this is the first score that rates AI outputs in drug-related queries with different aspects summed up. The following aspects have been investigated individually: completeness [4], references [4], correctness [5,27], accuracy [10,27], and possible harm to patients [4,5,10]. We designed AQUOS to be applied to other drug-related queries, not just renal dosage adjustment. As a few criteria have been similarly used in the studies mentioned above, there is a basis for external validity. Nevertheless, it should be tested in other areas, studies, and languages.

Although AQUOS has some weaknesses, such as the AI chatbots regularly losing points for not giving references at all—despite the prompt instructing them to do so—and for not providing good or accessible references, as long as the content and the recommendations are correct and accurate, there would typically be no harm to the patient. Therefore, it may be necessary to adjust AQUOS depending on the research question.

### Risks of AI-Generated Clinical Information

AI chatbots generate fluent and professionally worded responses that may appear authoritative regardless of their actual accuracy or completeness. Users may, therefore, treat chatbot outputs as expert recommendations rather than as an informational starting point that requires critical evaluation. A related issue is automation bias, in which health care professionals may have overconfidence in the AI-generated outputs due to their confident tone, which can lead to dosage decisions being made without adequate verification.

The recurring question of accountability for such clinical decisions remains largely unresolved. Regulatory frameworks addressing AI in high-risk settings are increasingly emerging internationally, with the European Artificial Intelligence Act [30] being a well-known example that explicitly classifies certain AI applications in health care as high risk and imposes requirements for transparency and human oversight. However, implementation remains variable across countries and health care systems, and the specific allocation of accountability in cases where AI-generated recommendations result in patient harm has not yet been adequately addressed in practice.

These considerations support the conclusion that AI chatbots should be positioned as informational assistance, with the understanding that final clinical responsibility remains with the responsible health care professional, particularly in complex scenarios such as renal dosage adjustment in geriatric patients.

### Clinical Role of AI Chatbots

The findings of this study should be interpreted within the context of a clearly defined scope of application. The AI chatbots evaluated here serve as informational aids and should not be mistaken for clinical decision support systems in the regulatory sense or as defined by medical device regulations. Appropriate use scenarios include support for educational purposes, preliminary orientation for clinicians, or self-checking in low-risk, nonurgent cases. AI chatbots are not suitable for directly guiding prescribing decisions, replacing pharmacist consultations, or operating without subsequent verification by a qualified health care professional. This distinction is particularly critical in high-risk settings, such as renal dose adjustment in geriatric patients, where errors can directly harm patients. Any integration of AI chatbot outputs into clinical workflows must, therefore, be accompanied by explicit precautions that ensure the final clinical decision remains with the responsible health care professional.

### Limitations

This study has some limitations. First, we used a uniform input structure, varying the GFR values and drug dosages. This approach ensured consistency across the cases, though it does not capture the potential variability that may arise from alternative prompt formulations or interaction types. While we did not incorporate few-shot examples, follow-up dialogs, adjustment settings such as temperature or system instructions, or the usage of the web interface instead of an application programming interface—which reduces reproducibility—our method was intended to reflect a common practical usage scenario. Since the rater was not blinded during scoring, the possibility of bias cannot be ruled out.

The patient data were from a single hospital, and we focused on the discharge medication. Our analysis concentrated specifically on the GFR and related dosage adjustments, which are clinically relevant, though other aspects of the medication could also be considered in future research.

These methodological choices, while deliberate, mean that the findings should be interpreted within the context of the study design. Future studies could expand the range of clinical variables, use diverse data sources, and explore different interaction modes and types to further evaluate chatbot performance.

### Comparison With Prior Work

Besides pharmaceutical or drug-related queries, there is an increasing number of scientific studies evaluating medical chatbots in health care.

Huo et al [31] conducted a systematic review to examine the aspects investigated in studies evaluating AI chatbots in health care contexts. They found that almost two thirds of the 137 included studies evaluated AI chatbots using subjective parameters only. They also noted a lack of detailed descriptions of prompt engineering, as well as insufficient consideration of patient safety, regulatory issues, and ethical considerations. Our study directly addresses several of the gaps identified by Huo et al [31], including the need for objective assessment and methodological transparency, such as in prompt design and safety or harm assessment. Complementary to this, other research has focused on evaluating conversational AI chatbots with particular attention to their limitations and associated concerns [32]. Wang et al [32] included 65 studies in their systematic review in which AI chatbots like ChatGPT were applied in health care contexts. Nearly half of the reviewed studies examined medical knowledge inquiries and reported a rather high precision in the AI chatbots’ responses. In 85% of all papers analyzed, concerns such as the reliability and bias of the AI chatbots were raised [32]. Our findings align with these results, as our evaluation using AQUOS and, for example, the study design with 2 different prompting times also places a strong emphasis on reliability.

Li et al [33] compared 8 different AI chatbots on 48 clinical questions using a combination of an expert panel and a 6D evaluation framework. They found that all the chatbots showed limitations and weaknesses in complex cases but also highlighted the potential of AI chatbots, always considering the associated risks [33]. Similarly, our study revealed such limitations, especially in more complex cases, such as those involving reduced renal function or multiple concomitant medications. Additionally, another review found that LLMs can support clinicians in various tasks but are not yet reliably applicable across all clinical domains, making deployment challenging [34].

### Conclusions

We developed and validated a new score (AQUOS) to examine the quality of AI chatbot answers for dose adjustment in patients according to their renal function. Using real patient data from a geriatric setting, we found that the quality of the AI chatbots’ responses varied depending on the chatbot, with values ranging from −19.0% to 95.2%. First, these responses were language-dependent. This is relevant because, in inpatient and outpatient settings, information is often sought in the patient’s native language. Furthermore, the results were highly reproducible and did not differ significantly at 2 points in time with independent search queries. However, it was found that the quality of the queries and the potential harm were adversely affected when kidney function decreased and the number of medications increased. In general, however, the quality of the responses is not yet convincing, especially in complex situations where advice from a chatbot is particularly sought after. ChatGPT proved to be the best chatbot in terms of quality. Future studies should evaluate AI chatbot performance using broader, more individualized clinical contexts to assess and compare directly whether additional information about the patients improves the accuracy and safety of AI-generated dosing recommendations. Further research should also be encouraged to find ways to influence the results obtained to make them suitable for practical use. In addition, ethical and legal issues surrounding the use of chatbots with real patients in treatment routines still need to be clarified in the future.

## Supplementary material

10.2196/87803 Multimedia Appendix 1 The artificial intelligence quality output score (AQUOS).

10.2196/87803 Multimedia Appendix 2 Artificial intelligence (AI) chatbot outputs and scoring examples.

10.2196/87803 Multimedia Appendix 3 Validation procedure of artificial intelligence quality output score (AQUOS).

10.2196/87803 Multimedia Appendix 4 Overall output scores (artificial intelligence quality output score [AQUOS]) of each artificial intelligence (AI) chatbot in German and English.

10.2196/87803 Multimedia Appendix 5 Correlation of the overall output scores (artificial intelligence quality output score [AQUOS]) of each artificial intelligence (AI) chatbot with glomerular filtration rate (GFR) categories and complexity categories in German and English, correlation coefficient (*P* value, double-sided).

10.2196/87803 Multimedia Appendix 6 Overall output scores (artificial intelligence quality output score [AQUOS]) of each artificial intelligence (AI) chatbot in German and English.

10.2196/87803 Checklist 1 CHART checklist.

## Abbreviations

AI: artificial intelligence
AQUOS: artificial intelligence quality output score
CHART: Chatbot Assessment Reporting Tool
GFR: glomerular filtration rate
LLM: large language model
WHO: World Health Organization
