# Supplementary material for: AI Chatbot Answers for Drug Dosing Adjustments According to Renal Function in Geriatric Patients Using the New Scoring System (AI Quality Output Score): Cross-Sectional Study
Source: JMIR AI. 2026 Jun 5;5:e87803. doi: 10.2196/87803 (PMC13240796; doi:10.2196/87803)
Supplement: Multimedia Appendix 1 [file ai-v5-e87803-s001.doc]

# Multimedia Appendix

Table S1 AI Quality Output Score (AQUOS)

| **Criteria** | **Criteria number** | **Questions for evaluation** | **Scoring** | **Notes and further information** |
| --- | --- | --- | --- | --- |
| **Completeness of drugs** | 1 | Can all drugs mentioned in the input be found in the output? | None of the drugs: 0 points  Less than half of the drugs: 1 point  Half of the drugs: 2 points  More than half of the drugs: 3 points  All drugs: 4 points | These first two criteria are solely based on what is mentioned in the output—there is no content evaluation at this point in the score. |
| **References** | 2 | Are references given for each drug? | No: 0 points  Less than half: 1 point  One half: 2 points  More than half: 3 points  Yes: 4 points |
| **Suitability of the references given** | 3 | Are the references up-to-date, reliable, adequate for the query, and explicit, e.g., for the specific drug and publicity accessible? | No: 0 points  Less than half of the references are appropriate: 1 point  Half of the references are appropriate: 2 points  More than half of the references are appropriate: 3 points  All of the references are appropriate: 4 points | Links work; adequate website for general use for the query (e.g., dosing.de, summaries of product characteristics, guidelines, original publications, European Medicine Agency…). For each information, an appropriate reference is given. No general links should be provided, e.g., [www.fachinfo.de](http://www.fachinfo.de/), but specifically for the relevant drug.  Is the reference/database freely accessible? |
| **Correct dose recommendation** | 4 | Is the dosage recommendation right according to approved sources, such as summaries of product characteristics or guidelines? | No: 0 points  Less than half is correct: 1 point  One half is correct: 2 points  More than half is correct: 3 points  Yes: 4 points |  |
| **Accuracy of the dosing advice** | 5 | How precise and accurate is the answer/ the output? | Inaccurate: 0 points  Partly accurate: 1 point  One half is accurate: 2 points  More than half is accurate: 3 points  Accurate: 4 points | Are specific dosage specifications provided, e.g., is ‘reduced’ used without a precise specification?  If the dosage has already been adjusted to the patient's GFR, but the recommendation is to reduce it to the dose where it already is, it has to be ranked as ‘inaccurate’ for this part of the output. |
| **Referral to medical/ pharmaceutical professionals or follow-up checks** | 6 | Does the output refer to medical or pharmaceutical professionals or follow-up checks? | No: 0 points  Yes: 1 point | Mentioning physicians, pharmacists, pharmacologists, and healthcare professionals as contact people. Reference to follow-up checks, monitoring, individual patient cases, etc. |
| **Additional information** | 7 | Is additional information provided that is not required to answer the query? | No: 0 points  Partly: -1 point  Half of the reply: -2 points  Mostly: -3 points  The complete reply: -4 points | E.g., definitions of the GFR, indications of the drugs, structural formulas, elimination route, etc. |
| **Medical expertise** | 8 | Are medical-specialised terms used in the wrong context? | No: 0 points  Partly: -1 point  Half of the reply: -2 points  Mostly: -3 points  Yes: -4 points |  |
| **Inappropriate words or phrases** | 9 | Are inappropriate words or phrases used? | No: 0 points  Partly: -1 point  Half of the reply: -2 points  Mostly: -3 points  Yes: -4 points | Software ‘dropout‘; chatbot replies in a different language than the one given in the input. |
| **Other** | | The score is split into two parts: first into positive criteria and then into negative criteria. | | |
| **Quality score (9 criteria)** | | Maximum 21 points, minimum: -12 points | | |
