# Supplementary material for: AI Chatbot Answers for Drug Dosing Adjustments According to Renal Function in Geriatric Patients Using the New Scoring System (AI Quality Output Score): Cross-Sectional Study
Source: JMIR AI. 2026 Jun 5;5:e87803. doi: 10.2196/87803 (PMC13240796; doi:10.2196/87803)
Supplement: Multimedia Appendix 2 [file ai-v5-e87803-s002.doc]

# Multimedia Appendix

Table S2 Overall output scores (AQUOS) of each AI chatbot in German and English, scores in mean (SD) in %; Renal function in four categories (1 – normal renal function, 2 – slightly reduced renal function, 3 – moderately reduced renal function, 4 – severely reduced renal function); Ger. = German, Eng. = English

| in % | Number of patients | ChatGPT | | Copilot | | Gemini | | scite | |
| --- | --- | --- | --- | --- | --- | --- | --- | --- | --- |
|  |  | Ger. | Engl. | Ger. | Engl. | Ger. | Engl. | Ger. | Engl. |
| Category 1 | 5 | 72.4% (10.8%) | 81.0% (18.6%) | 73.3% (14.2%) | 77.1% (6.8%) | 30.5% (67.7%) | 56.2% (22.7%) | 69.5% (7.8%) | 73.3% (5.8%) |
| Category 2 | 62 | 72.5% (11.6%) | 77.3% (11.2%) | 72.0% (11.2%) | 74.6% (8.8%) | 40.3% (29.7%) | 48.3% (33.6%) | 66.7% (14.5%) | 71.8% (6.5%) |
| Category 3 | 25 | 71.0% (12.7%) | 74.1% (12.7%) | 70.5% (10.5%) | 69.3% (16.0%) | 36.0% (41.3%) | 41.9% (60.0%) | 65.0% (13.2%) | 66.9% (11.7%) |
| Category 4 | 8 | 63.7% (19.6%) | 73.2% (14.3%) | 70.2% (11.3%) | 71.4% (9.4%) | 28.0% (49.4%) | 45.8% (31.4%) | 60.1% (26.8%) | 66.1% (14.6%) |
