# Supplementary material for: AI Chatbot Answers for Drug Dosing Adjustments According to Renal Function in Geriatric Patients Using the New Scoring System (AI Quality Output Score): Cross-Sectional Study
Source: JMIR AI. 2026 Jun 5;5:e87803. doi: 10.2196/87803 (PMC13240796; doi:10.2196/87803)
Supplement: Multimedia Appendix 3 [file ai-v5-e87803-s003.docx]

# Multimedia Appendix

## Validation Procedure of AQUOS

Phase 1: Internal Validation
In the first phase, the reliability and internal validity of AQUOS was assessed. A panel of two licensed pharmacists independently evaluated the AI chatbot answers of 10 patient cases using AQUOS (Multimedia Appendix 1). In cases of disagreement, a third pharmacist acted as adjudicator to establish consensus. Inter-rater reliability was quantified using Cohen’s Kappa coefficient (20), a statistical measure of agreement beyond chance. A strong level of agreement (κ > 0.8) was interpreted as evidence of objectivity and replicability, supporting the use of a single rater for subsequent evaluations.

Phase 2: External Validation
For external validation, an independent panel of four licensed pharmacists – serving as external experts and representing the reference standard (gold standard) – evaluated the same 10 AI chatbot answers of the 10 patient cases. Each chatbot-generated response was rated on a five-point Likert scale reflecting clinical appropriateness and quality. Agreement among experts was assessed using intraclass correlation coefficient (ICC) (21). To establish criterion validity, the median expert ratings were correlated with the corresponding AQUOS scores using Spearman’s correlation. A significant positive correlation was interpreted as evidence that AQUOS validly reflects expert judgement.
