# Supplementary material for: AI Chatbot Answers for Drug Dosing Adjustments According to Renal Function in Geriatric Patients Using the New Scoring System (AI Quality Output Score): Cross-Sectional Study
Source: JMIR AI. 2026 Jun 5;5:e87803. doi: 10.2196/87803 (PMC13240796; doi:10.2196/87803)
Supplement: Multimedia Appendix 5 [file ai-v5-e87803-s005.doc]

# Multimedia Appendix

Table S3 Correlation of the overall output scores (AQUOS) of each AI chatbot with GFR Categories and Complexity Categories in German and English, Correlation-Coefficient (p-value, double-sited); Ger. = German, Eng. = English

|  | Number of patients | ChatGPT | | Copilot | | Gemini | | scite | |
| --- | --- | --- | --- | --- | --- | --- | --- | --- | --- |
|  |  | Ger. | Engl. | Ger. | Engl. | Ger. | Engl. | Ger. | Engl. |
| GFR Categories | 100 | -0.156 (0.122) | -0.215 (0.032) | -0.098 (0.330) | -0.258 (0.009) | -0.171 (0.090) | -0.168 (0.095) | -0.184 (0.067) | -0.357 (0.000) |
| Complexity Categories | 100 | -0.157 (0.120) | -0.145 (0.150) | -0.137 (0.174) | -0.077 (0.446) | -0.198 (0.048) | -0.167 (0.097) | -0.356 (0.000) | -0.239 (0.016) |
