# Supplementary material for: AI Chatbot Answers for Drug Dosing Adjustments According to Renal Function in Geriatric Patients Using the New Scoring System (AI Quality Output Score): Cross-Sectional Study
Source: JMIR AI. 2026 Jun 5;5:e87803. doi: 10.2196/87803 (PMC13240796; doi:10.2196/87803)
Supplement: Multimedia Appendix 6 [file ai-v5-e87803-s006.doc]

# Multimedia Appendix

Table S4 Overall output scores (AQUOS) of each AI chatbot in German and English, scores in mean (SD) in %; Complexity of medication in 3 categories (1 –low complexity, 2 – medium complexity, 3 – high complexity); Ger. = German, Eng. = English

| in % | Number of patients | ChatGPT | | Copilot | | Gemini | | scite | |
| --- | --- | --- | --- | --- | --- | --- | --- | --- | --- |
|  |  | Ger. | Engl. | Ger. | Engl. | Ger. | Engl. | Ger. | Engl. |
| Low complexity | 33 | 73.2% (10.0%) | 79.1% (9.0%) | 73.2% (10.1%) | 74.3% (11.0%) | 40.4% (30.9%) | 48.2% (46.8%) | 70.4% (7.5%) | 71.9% (8.3%) |
| Medium complexity | 46 | 71.7% (12.0%) | 76.0% (11.6%) | 71.6% (10.0%) | 72.3% (11.7%) | 38.3% (33.8%) | 47.6% (39.0%) | 65.0% (16.8%) | 70.4% (7.9%) |
| High complexity | 21 | 68.0% (17.3%) | 72.8% (17.1%) | 68.9% (14.3%) | 73.2% (10.4%) | 32.4% (49.9%) | 43.3% (25.5%) | 60.5% (17.4%) | 67.1% (12.1%) |
