# Supplementary material for: AI Chatbot Answers for Drug Dosing Adjustments According to Renal Function in Geriatric Patients Using the New Scoring System (AI Quality Output Score): Cross-Sectional Study
Source: JMIR AI. 2026 Jun 5;5:e87803. doi: 10.2196/87803 (PMC13240796; doi:10.2196/87803)
Supplement: Checklist 1 [file ai-v5-e87803-s007.pdf]

The CHART Checklist

HEADING	#	CHART CHECKLIST ITEM	Page #*
**Title & Abstract**			
**Title**	**1a**	State that the study is assessing one or more generative AI-driven chatbots for clinical evidence or health advice.	1
**Abstract/Summary**	**1b**	Apply a structured format, if applicable.	1
**Introduction**			
**Background**	**2a**	State the scientific background, rationale, and healthcare context for evaluating the generative AI-driven chatbot(s), referencing relevant literature when applicable.	2
	**2b**	State the aims and research questions including the target audience, intervention, comparator(s), and outcome(s).	2
**Methods**			
**Model Identifiers**	**3a**	State the name and version identifier(s) of the generative AI model(s) and chatbot(s) under evaluation, as well as their date of release or last update.	3
	**3b**	State whether the generative AI model(s) and chatbot(s) are open-source or closed-source/proprietary.	3
**Model Details**	**4a**	State whether the generative AI model was a base model or a novel base model, tuned model, or fine-tuned model.	3
	**4b**	If a base model is used, cite its development in sufficient detail to identify the model.	3
	**4c**	If a novel base model, tuned model, or fine-tuned model is used, describe the pre- and/or post-implementation/deployment data and parameters.	-
**Prompt Engineering**	**5a**	Describe the evolution of study prompt development.	2, 3
	**5ai**	Describe the sources of prompts.	2, 3
	**5aii**	State the number and characteristics of the individual(s) involved in prompt engineering.	2, 3
	**5aiii**	Provide details of any patient and public involvement during prompt engineering.	2, 3
	**5b**	Provide study prompts.	3, #2
**Query Strategy**	**6a**	State route of access to generative AI model.	3
	**6b**	State the date(s) and location(s) of queries for the generative AI-driven chatbot(s) including the day, month, and year as well as city and country.	2
	**6c**	Describe whether prompts were input into separate chat session(s).	3
	**6d**	Provide all generative AI-driven chatbot output/responses	10
**Performance Evaluation**	**7a**	Define the ground truth or reference standard used to define successful generative AI-driven chatbot performance.	3
	**7b**	Describe the process undertaken for generative AI-driven chatbot performance evaluation.	3, #3
	**7bi**	State the number and characteristics of team members involved in performance evaluation.	3
	**7bii**	Provide details of any patients and public involvement during the evaluation process.	3
	**7biii**	State whether evaluators were masked to the identity of the generative AI-driven chatbot(s) under assessment.	9

**Sample Size**	**8**	Report how the sample size was determined.	2
**Data Analysis**	**9a**	Describe statistical analysis methods, including any evaluation of reproducibility of generative AI-driven chatbot responses.	3, 4
	**9ai**	Report the measures used for performance evaluation.	3, 4
**Results**			
	**10a**	Report the alignment between generative AI-driven chatbot output and ground truth or reference standard using quantitative or mixed methods approaches as applicable.	4-7
	**10b**	For responses deviating from the ground truth or reference standard, state the nature of the difference(s).	4-6
	**10c**	Report the assessment for potentially harmful, biased, or misleading responses.	6
**Discussion**			
	**11a**	Interpret study findings in the context of relevant evidence.	7-9
	**11b**	Describe the strengths and limitations of the study.	8, 9
	**11c**	Describe the potential implications for practice, education, policy, regulation, and research.	8-10
**Open Science**			
**Disclosures**	**12a**	Report any relevant conflicts of interest for all authors.	10
**Funding**	**12b**	Report sources of funding and their role in the conduct and reporting of the study.	10
**Ethics**	**12c**	Describe the process undertaken for ethical approval.	2
	**12ci**	Describe the measures taken to safeguard data privacy of patient health information, as applicable.	2, 3
	**12cii**	State whether permission/licensing was obtained for the use of original, copyrighted data.	2
**Protocol**	**12d**	Provide a study protocol.	-
**Data availability**	**12e**	State where study data, code repository, and model parameters can be accessed.	10

*If in supplementary appendix, indicate "supp" and appendix #, if applicable.
